# The RoScan Thermal 3D Body Scanning System: Medical Applicability and Benefits for Unobtrusive Sensing and Objective Diagnosis

**DOI:** 10.3390/s20226656

**Published:** 2020-11-20

**Authors:** Adam Chromy, Ludek Zalud

**Affiliations:** 1 CEITEC—Central European Institute of Technology, Brno University of Technology, Purkynova 656/123, 612 00 Brno, Czech Republic; ludek.zalud@ceitec.vutbr.cz; 2 Faculty of Electrical Engineering and Communications, Brno University of Technology, Technicka 3082/12, 616 00 Brno, Czech Republic

**Keywords:** multimodal imaging, multispectral imaging, 3D thermography, high-accuracy 3D scanning, robotic 3D scanning

## Abstract

The RoScan is a novel, high-accuracy multispectral surface scanning system producing colored 3D models that include a thermal layer. (1) Background: at present, medicine still exhibits a lack of objective diagnostic methods. As many diseases involve thermal changes, thermography may appear to be a convenient technique for the given purpose; however, there are three limiting problems: exact localization, resolution vs. range, and impossibility of quantification. (2) Methods: the basic principles and benefits of the system are described. The procedures rely on a robotic manipulator with multiple sensors to create a multispectral 3D model. Importantly, the structure is robust, scene-independent, and features quantifiable measurement uncertainty; thus, all of the above problems of medical thermography are resolved. (3) Results: the benefits were demonstrated by several pilot case studies: medicament efficacy assessment in dermatology, objective recovery progress assessment in traumatology, applied force quantification in forensic sciences, exact localization of the cause of pain in physiotherapy, objective assessment of atopic dermatitis, and soft tissue volumetric measurements. (4) Conclusion: the RoScan addresses medical quantification, which embodies a frequent problem in several medical sectors, and can deliver new, objective information to improve the quality of healthcare and to eliminate false diagnoses.

## 1. Introduction

In general terms, the effectiveness of treatment, and thus also the recovery time and the amount of resources spent, stands on two prominent pillars: correct diagnosis and appropriate therapy [1]. At present, the latter pillar is comparatively well-characterized thanks to the availability of a wide range of knowledge sources generated through simplified global communication. Recommended therapies are often standardized, with the “best practice” methods systematically presented in research articles. Moreover, and importantly in the given context, almost any diagnosis can be paired with a suitable treatment approach [2].

The pace of progress in the former pillar, however, is markedly different. Although the correct diagnosis is at least as important as proper therapy, many of the current, commonly used diagnostic procedures still rely on healthcare professionals’ experience, intuition, and memory. The correctness of the decision then depends on the specific abilities of the medical specialist [3]. The diagnosis can be very close to the patient’s real condition, but it can also be completely wrong. The variance of the results then embodies a major problem within intuitive medicine.

To prevent mistakes in determining the correct diagnosis, to decrease the required time, and to reduce the dependence of successful disease classification on the doctor’s expertise and experience, supporting diagnostic medical devices are introduced increasingly more often. Most hospitals are presently equipped with general-purpose imaging methods, such as X-ray, MRI, and CT; these tools, however, may be very expensive and thus less accessible to smaller medical centres. In such cases, smaller, cheaper, and more specialized instruments are employed.

**Figure 1:**
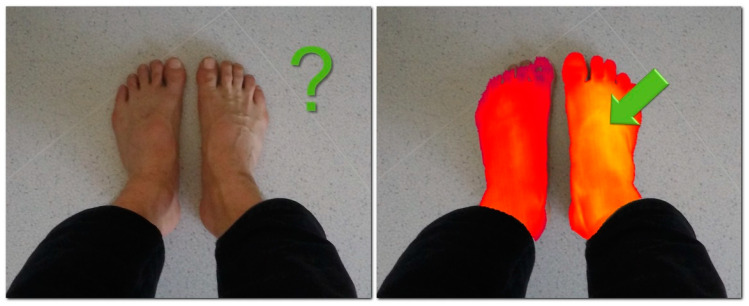
The symptoms have not progressed far enough to be observable (**left**), but the inflammation is already clearly visible in the thermal image (**right**).

Almost all types of injuries, multiple diseases, and pathological changes share a significant symptom: the local temperature change. This thermal abnormality is caused by increased blood flow and stronger cellular metabolism in the affected area; the two aspects then induce a local temperature difference generated by the given negative health-related effect [4]. Such local thermal deviations can be detected and visualized via thermal cameras operating within the long-wave infrared spectrum (LWIR) [5]. As the resolution of thermal imagers is about 0.04 °C [6], even the smallest changes are detectable, and the disease may be terminated before developing significantly enough to produce observable symptoms like redness or swelling (Figure 1).

**Figure 2:**
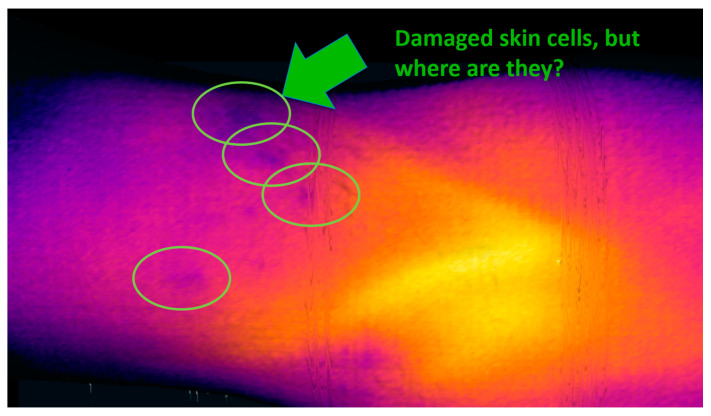
The thermal footprints cannot be clearly assigned to particular locations on the body. The lack of sharp edges and other spot landmarks makes exact localization in a single 2D thermal image impossible unless registration is performed with such a supplementary layer that yields clear features.

**Figure 3:**
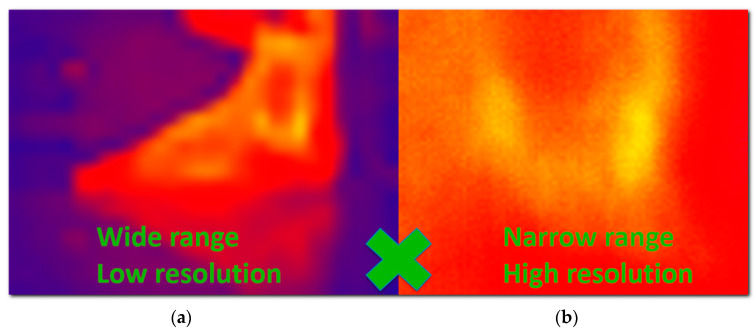
The impossibility of simultaneously reaching both a wide range and a high resolution restrains the use of thermal imaging in medicine: (**a**) in a wide-range view, the whole body part is visible, but the details of the temperature distribution are not; (**b**) in a narrow-range view, the details are visible, but the region cannot be localized, and context characterizing the whole affected area is missing.

Medical thermography is generally referred to as Digital Medical Thermal Imaging (DMTI); the technique currently finds use in several relevant applications (e.g., inflamed tissue analysis [7,8], cancer detection [9], blood perfusion [10], and human stress research [11]), although it has not been developed sufficiently to date. The main factors limiting the usability and popularity of this powerful imaging approach consist of three central drawbacks [12,13,14,15,16]:

1. Exact localization problem—due to the lack of easily recognizable and clearly bounded landmarks in the thermal image (which contains mostly smooth gradients instead of sharp edges), it is not possible to determine the exact position of a thermal deviation on the skin. This issue is illustrated in Figure 2, namely, the indicated spots with a locally lower temperature; however, we are presently unable to accurately assign the individual points to exact places on the body, and it is then impossible to distinguish whether such thermal footprints relate to, for example, an area of eczema or a birthmark in the vicinity.
2. Resolution vs. range problem—the low resolution of thermal imagers forces the user to search for a compromise between two options: (a) a wide-range view, where the entire part of the body is represented, but the resolution is too low to show enough details (Figure 3a); and (b) a detailed view providing sufficient resolution but containing a very narrow segment of the body, from which the context and exact position are difficult to understand (Figure 3b). As the thermogram analysis consists predominantly of comparing the temperature in body regions, the clinician must see a relatively large area of the body; simultaneously, the high resolution has to be preserved to deliver the required details. Overall, the resolution vs. range problems is one of the most limiting drawbacks restricting the development of DMTI.
3. Qualitative only, no quantification—although 2D thermography basically constitutes a quantification tool (since it accurately measures the temperature in each pixel), in terms of the medical quantification of disease or injury it is a qualitative method only [13,17]. By using it, we can recognize the physiological and non-physiological regions of the body but remain unable to quantify them. According to [13], this issue stems from the following aspects: (1) changes in the thermal image are gradual and continuous, and thus the image contains very few clear landmarks (sharp edges or clear points), which are required for specific definition of the region of interest (ROI). The exactly bounded ROI embodies a necessary precondition to enable quantification within the impacted region. (2) The DMTI output is created by the lossy transformation of the real 3D world into a 2D projection; thus, the image is distorted. This scenario does not allow us to implement certain steps, for example, measuring the surface of the affected area: we can see only the projection, which is significantly influenced by the actual setup of the camera in relation to the scanned body. (3) In order to obtain two comparable thermograms, we have to exactly preserve the scene, including the exact position of the subject (as even tiny changes of the position may exert a substantial impact on the results). Such a condition is hardly achievable in currently used setups.

The RoScan multispectral 3D scanning project, which is described in the following chapters, addresses and eliminates all of the three DMTI problems outlined above. Thus, the novel system upgrades DMTI to wide-range high-accuracy 3D thermography, which can be readily used as an objective quantitative tool in various medical subdomains [10].

At present, the actual medical quantification embodies a topic broadly discussed throughout the healthcare sector. Insufficiently accurate, inadequately sensitive, or excessively subjective diagnostic methods are perceived as very restrictive in several medical domains, including dermatology, traumatology, physiotherapy, and forensic sciences.

In dermatology, coarse and subjective scoring systems remain in wide use as the primary means of evaluation because an objective and reliable in vivo quantification method applicable to a particular region of interest has not been developed to date. Due to this deficiency, a diagnosis based on such subjective data is then unable to reveal tiny abnormalities to detect the disease in its early stage.

Traumatology and forensic sciences would greatly welcome an objective method for quantifying and monitoring diverse effects or symptoms, such as bruises and their severity; however, this type of method is still missing even in the two fields.

In physiotherapy, one of the common symptoms is a change in the body volume. These manifestations have to be measured objectively and sensitively in order to reveal an emerging disease already at the initial stage. Although the changes are currently measurable in a comparatively accurate manner, we are unable to distinguish between a physiological (e.g., muscle growth) and a non-physiological (e.g., swelling) change. Moreover, it is also difficult to assess the impact of the treatment procedures because the existing treatment evaluation exploits subjective health surveys or low-resolution scoring systems with poor inter-observer correlation.

These problems (and many others) are tackled by the RoScan multispectral 3D scanning system. In this study, the basic principles and benefits of the approach are characterized and demonstrated on several medical applications where pilot case studies were performed with positive results.

## 2. Materials and Methods

This paper discusses novel approaches to and applications within 3D thermal scanning. The presented scanning system, developed completely at Brno University of Technology, combines a high operational flexibility with high absolute accuracy of the resulting 3D model. Such a fusion of capabilities is still uncommon in current commercial 3D scanners, as these usually embody a compromise between the scanning accuracy and flexibility [18]; in medicine, however, both of these parameters are needed. A flexible and accurate 3D surface scanning system is thus very likely to find wide use in various medical domains, especially if it yields extensive 3D models and maintains the high accuracy across all the spectral layers, thus providing options not regularly available within the state of the art.

**Figure 4:**
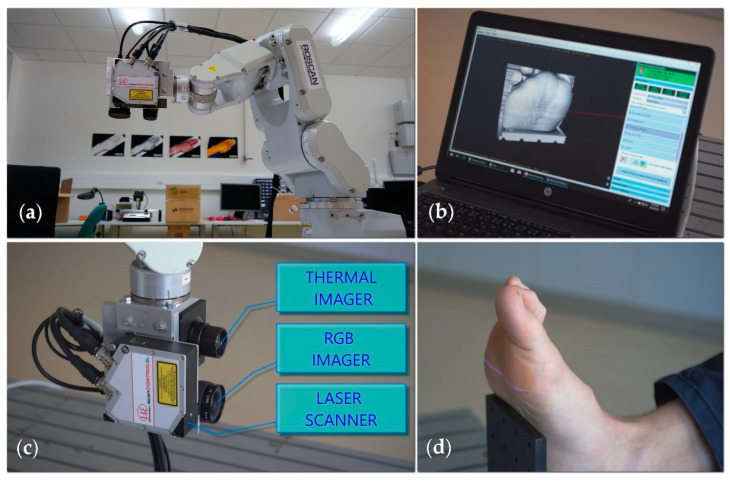
The RoScan system: (**a**) the main hardware components, namely, the robotic manipulator and head with several sensors; (**b**) the software yields a 3D mesh in real time; (**c**) the sensing head comprises a 2D laser scanner, a thermal imager, and an RGB camera; (**d**) the sensing head moves along the body, capturing thousands of distance profiles needed to create a 3D spatial model and taking several images from the thermal and visible spectra; the spectra are then mapped onto the surface.

The RoScan multispectral 3D system consists of a robotic manipulator and several sensors, grouped together at the endpoint into a sensing head (Figure 4a). To allow medical use, we combine a laser scanner, a color camera (wavelength range between 390 and 750 nm), and a thermal imager (wavelength range 8 to 14 µm); however, the system is modular, meaning that the cameras can be replaced by other sensors providing data in various spectral ranges.

The manipulator moves the sensors along the body being scanned (Figure 4d), and the software processes, in real time, the distance data measured by the laser scanner into the form of a 3D surface model (Figure 4b).

In addition to the laser scanner necessary for acquiring the 3D model itself, the robot’s endpoint is equipped with a color camera (Figure 4c), which delivers color information relating to each point of the 3D mesh; a thermal imager is also included (but will be discussed only later in the text).

To this point, the RoScan’s output may have seemed to be almost the same as that of commercially available 3D scanners; such an impression, however, will change quickly if we emphasize the fact that the output model can be large and complex without any influence on the accuracy. Compared to other current 3D scanning instruments, the robotic manipulator ensures very accurate and independent localization of the sensors; the accuracy of the resulting model then does not affect the object’s appearance or shape. The method is robust, and the accuracy constraints are identical regardless of the scanned scene. Smooth homogeneous structures or repeated patterns are processed flawlessly (this being a substantial difference from the problematic procedure that characterizes, for example, methods based on stereophotogrammetric principles [19]), and the 3D model does not exhibit a cumulative deviation (unlike the products of, for example, handheld scanners [20] or other methods based on Iterative closest point (ICP) principle [21,22,23]).

An additional information layer, unavailable in current 3D scanners, is generated by the thermal imager. The advantageously composed structure utilizes the robotic manipulator to capture the temperature images and 3D spatial data at different positions: The images are taken at a significantly greater proximity to compensate for the low resolution of thermal cameras and to achieve the same resolution in the thermogram, the other layers, and the entire 3D model. Consequently, a true, non-interpolated temperature value is assignable to each model point, even in extensive and highly detailed models. This capability then resolves and eliminates one of the problems that accompany the use of DMTI (see Section 1 and Section 2.4, issue No. 2, resolution vs. range problem).

In the RoScan, the output is a multispectral 3D computer model providing a color shape coupled with the thermal distribution. The combination of 3D data, thermal data, and a color image addresses also the DMTI drawbacks Nos. 1 and 3, as further explained in Section 2.4.

This paper briefly exposes the abilities, functions, and benefits of the entire RoScan system, focusing on its applicability; multiple details on the implementation are then outlined in other papers written by the authors of this article. The studies in question are dedicated to the actual 3D scanning [24,25,26], the mapping of 2D images onto the surface of the 3D model [27], and the laser scanner and camera calibration [28,29].

### 2.1. Capturing 3D Models with the RoScan

A multispectral 3D model is captured via the robotic manipulator moving the sensing head around the scanned object and along the predefined trajectory. The standard approach consists in defining different trajectories for each body part to be scanned; such predefined trajectories are simply selected according to the body part where a disease is expected.

**Figure 5:**
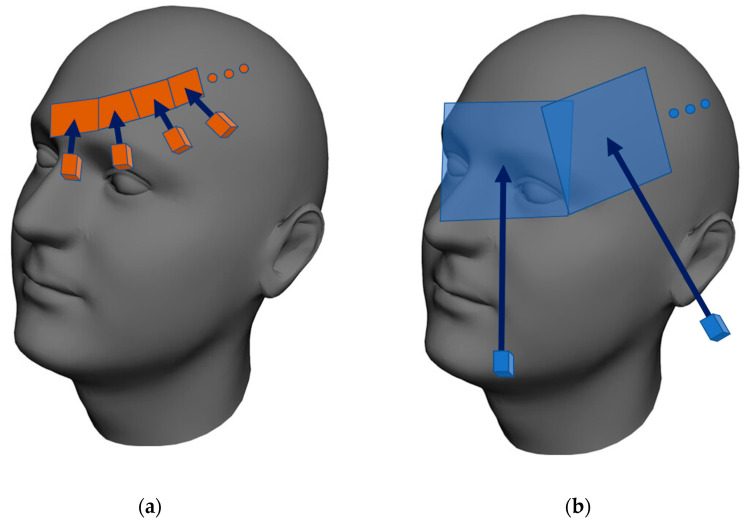
The procedures of reaching the same resolution rates in all of the spectral layers despite the native resolution of the sensors differ significantly: (**a**) hundreds of thermal images are captured at a very short distance, yielding a high resolution in a single image; the range, however, is narrow. Multiple images are taken and then merged by mapping onto the surface to form a single, high-resolution layer covering the whole model; (**b**) the native resolution of the color camera is markedly higher, meaning that only several images taken at a greater distance are needed to reach the final resolution identical with that achieved in scenario (**a**).

During the execution of a trajectory, the laser scanner captures the distance profiles, while the cameras collect the thermal and color images. The color images and distance profiles are obtained at similar proximities because both sensors exhibit an almost identical resolution rate. The resolution of the thermal imager is nevertheless significantly lower, making us capture (compared to the corresponding process performed with the color camera) markedly more thermal images from a significantly shorter distance. Thus, we finally obtain the same resolution in the color and the thermal layers (Figure 5).

All of the collected distance profiles are aligned to the same system of coordinates by using homogeneous transformations; subsequently, the 3D shaded mesh is generated [25].

In the next step, the 2D images from the cameras are mapped to the surface of the 3D model [27]. At this stage, the ray-tracing algorithm [30] is employed to examine the visibility of each point of the mesh from the camera. If a single point is visible in multiple images, the resulting temperature is defined as the average of the values, as follows: the color in such points is computed as linear interpolation between the colors from these images, weighted by the angle relative to the 3D surface normal because the lightness of the color is influenced by the light reflection angle.

The output model is delivered in a particular file format, which can be opened in the RoScan Analyzer software tool, described in the next section.

### 2.2. Tool to Allow 3D Model View and Analysis

Multispectral 3D surface models created with the RoScan can be displayed via the RoScan Analyzer. In addition to the common features, such as rotating, scaling, and adjusting the modes of view, the software instrument also facilitates measuring many spatial parameters of the selected region of interest (ROI) and allows exporting the 3D data in several standard file formats (PTS, XYZ, and PLY); the data are then processable with any third-party 3D modeling program.

**Figure 6:**
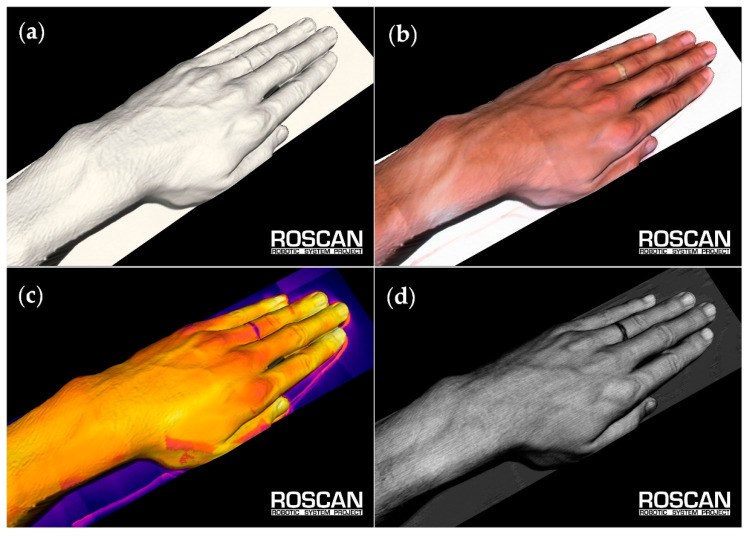
The output model of the RoScan system consists of several layers: (**a**) a 3D spatial representation without coloring; (**b**) a surface with true colors; (**c**) false-color-mapped thermal data; (**d**) false-color-mapped roughness data.

**Figure 7:**
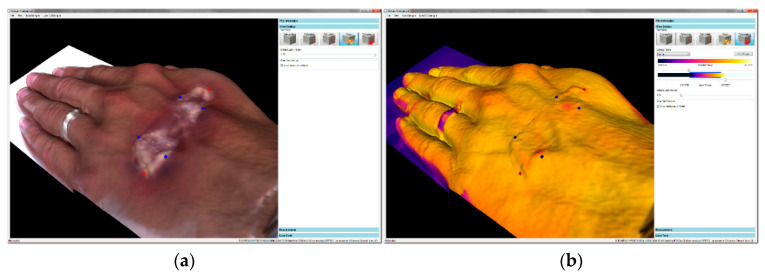
The navigation between several layers carried out by using points of interest (POIs): (**a**) selecting the area with suspicious features in the color layer; (**b**) examining the temperature at highlighted points in the thermal layer. In this case, we did not observe any thermal changes relating to the effect indicated in the image (**a**); the observation corresponds to reality, as the injury was created by a make-up artist.

Each model can be viewed in the following modes (Figure 6):

- Color—the basic view of the clinician. In the model, this layer is used mainly for localization, either by natural landmarks of the skin (such as pigmented spots), via pen-marked markers, or through the use of region boundaries; such landmarks can be highlighted to be visible also in other layers (Figure 7). Alternatively, when highlighting abnormal places in the visualized thermal layer, we can adopt the opposite approach, which relies on localizing the spots in a color representation.
- Temperature—the layer with false colors that correspond to the temperature of the skin; here, the exact value of a particular point can be examined by clicking. To reach the appropriate contrast between the physiological and the non-physiological areas, we can adjust the coloring palette by moving a slider.
- Roughness—this layer shows false colors that correspond to the roughness of the scanned surface. This output utilizes the measuring principle of the applied laser scanner, which exploits triangulation [31]. As a side result of this measuring method, the divergence of the reflected beam is acquired; such divergence correlates with the roughness of the scanned surface.
- Surface only—in certain cases, the coloring and shading of the model may hide some details or produce an optical illusion. Representing the surface in a single color allows us to see even the tiny details that normally often remain uncovered. When spatial details are to be examined, it is often convenient to show the 3D surface only, with the coloring eliminated.

On each layer, the user can select points of interest (POIs), facilitating spatial measurements with the model. In the selected points or regions of interest (ROIs) defined thereby, the following parameters can be assessed:

- Distance [mm]—separates several selected points. The distances are measured either in the direct mode or via the shortest path along the surface (these options are applicable in determining, e.g., the circumference of the ROI and the distance between the focus and the landmark).
- Angle [degree]—the angle between every three subsequent POIs, including, for example, those that allow an analysis of vertebrae positions.
- Surface area [mm^2^]—relating to the whole model or the ROI only, for example, the area of burn, or extent of lesion.
- Volume [mm^3^]—concerning the entire model or a part thereof. Such a portion can be defined by the cutting plane or deflected cutting surface. Useful for, e.g., measurements of the limb volume.
- Color—of a selected POI or the average color of the ROI. It is a more objective alternative to the currently used scoring systems for evaluating lesion redness.
- Roughness—defined by the beam divergence width (in the sensor pixels), which is the dimensionless index correlating with the surface roughness at a particular point. We can either examine such roughness or calculate the average roughness of the ROI.
- Temperature [°C]—of a highlighted point or the average temperature of the ROI. This parameter has the highest information value, since it correlates with inflammation in the area.

Such a multimodal 3D computer model finds use in, for example, inflammation monitoring. The process then comprises the following stages: first, the thermal layer is studied, and possible thermal abnormalities are highlighted in the 3D model. Second, we switch to the color mode, which facilitates exact localization of the inflammation foci and allows us to set up the region of interest (ROI). Afterwards, we can quantify the inflammation activity (usually in the form of the mean temperature within the ROI). At the last stage, the swelling-induced volume variation is measured accurately.

The inflammation monitoring and quantification constitute only one of the RoScan’s potential target applications. To illustrate the claim, we can note in this context that while inflammation leads to a positive thermal data gradient, ischemia results in a local decrease; thus, the RoScan may be employed in the monitoring of diabetic necrotic tissues.

### 2.3. Key Parameters of the RoScan

The central advantage of the discussed concept rests in the applied combination of spatial, color, and thermal data within a single 3D model; such an approach delivers new research opportunities, which would otherwise remain virtually impossible to implement. More concretely, the RoScan has a major potential in the objective evaluation of spatial changes of the body; this medical task includes, above all, the monitoring of oedemas, muscle growth and atrophy, inflammation, and necrosis.

The color layer guarantees the precise selection of a ROI (via natural landmarks or markers drawn on the subject’s skin). The thermal layer helps to find inflamed areas or determine if a spatial change is caused by physiological or non-physiological processes. Finally, 3D scanning yields an undistorted view, thus offering an effective alternative to standard 2D imaging technologies, where the distortion prevents objective measurements from being performed. The essential capabilities and properties of the RoScan system are summarized in Table 1.

The price of the device indicated in the table above relates to the research setup characterized herein; that is, a configuration which utilizes a robotic manipulator. Advantageously, this component delivers superior flexibility and versatility, allowing the entire device to find use in many different applications, even outside of medicine. If deployed in practice, however, the system is usually not required to perform an overly wide range of activities, and thus its six-degrees-of-freedom flexibility can become overkill. The manipulator can then be replaced by a cheaper (but still very accurate) positioning system with fewer degrees of freedom (Rails, Cartesian robots, etc.), maintaining the key parameters. As the price of the manipulator amounts to 19,000 EUR out of the total of 30,000 EUR, the acquisition costs could then drop markedly.

Although the purchase costs are significantly lower than those in the current imaging methods, the most extensive savings are achieved in regular operation, where the RoScan generates almost zero additional costs, regardless of the frequency of use. This is a major benefit compared to, for example, MRI, in which each scan is worth 200–400 EUR [32,33].

### 2.4. Eliminating the Drawbacks of DMTI

**Figure 8:**
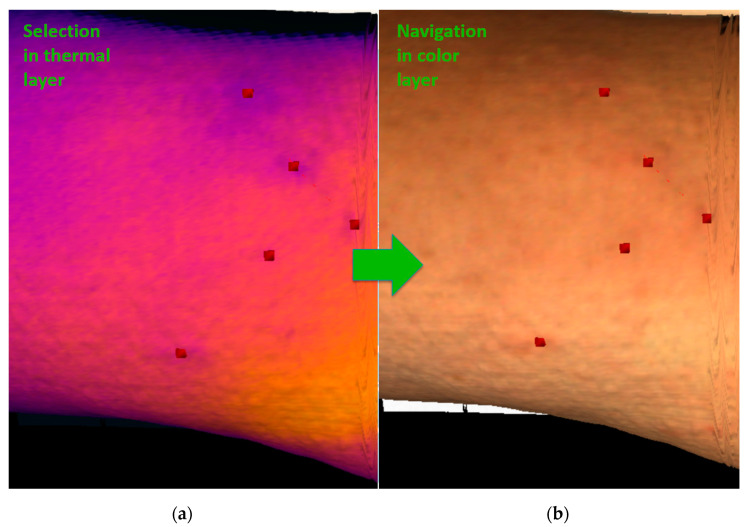
Resolving the exact localization problem: (**a**) the potentially problematic spots are highlighted in the thermal layer of the 3D model; (**b**) the selected points are localized in the color layer.

**Figure 9:**
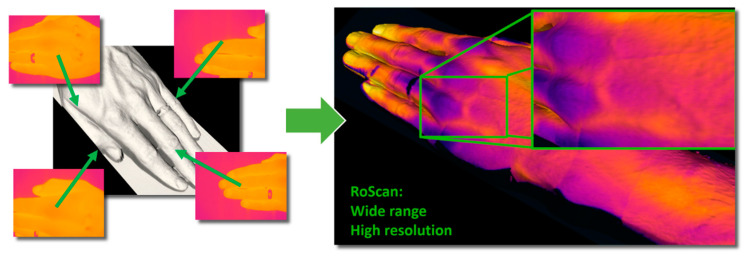
The resolution vs. range problem: combining multiple images with the RoScan delivers a wide range and a high resolution simultaneously.

As described in the Introduction, thermography is considered a useful method, even though its benefits are limited by three fundamental drawbacks [12,13,14,15,16]. These issues are addressed by the RoScan, as follows:

(1) The *exact localization problem* arises from a lack of clear point objects in a thermogram. Despite being able to detect areas with non-physiological behavior in a thermogram, we cannot determine exactly where they are (Figure 2 and Section 1, issue No. 1, the exact localization problem). For such a purpose, mutual registration of the thermal and the color layers in the RoScan appears to be advantageous, as the potentially problematic spots are marked in the thermal layer (Figure 8a) and then localized in the color one (Figure 8b), which comprises a significantly higher number of landmarks allowing sufficient orientation (e.g., pigmented spots, scars, eczema, and veins).

(2) The *resolution vs. range problem* is generated by the low resolution of thermal imagers. Since the thermal changes inside the human body are usually small and can hide in the physiological temperature gradients [8], it is necessary to provide data having very high resolution [13]. This objective can be achieved by focusing the thermal imager on the nearest possible distance; the obtained range of view, however, will be narrow and will neither contain enough information to perform the localization nor cover the whole area affected (Figure 3 and Section 1, issue No. 2, the resolution vs. range problem). The RoScan resolves the problem by capturing multiple images at a near distance and from a known position; these images are then mapped on the 3D surface, thus being in fact merged into a single, large, high-resolution image (Figure 9).

(3) The most prominent drawback lies in that classic DMTI is a mere qualitative tool, enabling us to distinguish between the physiological and non-physiological states of the body but lacking the ability to quantify them [13,17]. This deficiency is eliminated through the data fusion ensured by the RoScan, where the advantageous combination of sensors delivers color 3D surface scans with hi-resolution thermal information (Figure 6). Thanks to the color layer, the ROI can be selected precisely (see Section 2.4, issue 1, the localization problem); such a step is unfeasible with DMTI. The 3D model features the ability to capture undistorted measurements and provides the data in a form independent from the point of view adopted in the scanning. Finally, the thermal layer exposes non-physiological areas and yields a quantifiable index reflecting the severity of the disease.

Resolving these limitations opens the door to suitable thermal imaging applications within the medical domain. Later in the text, a set of pilot case studies is presented, showing the potential of quantification carried out via 3D thermal imaging.

## 3. Results

Several pilot experiments were performed within multiple medical specialties to validate the benefits of the RoScan. As this is the first application of 3D thermography utilizing the RoScan in the discussed field (and, in some instances, even thermography itself), we preferred case studies to broader analyses with wider populations of subjects. The outcomes of these experiments pointed to considerable usability of the device in medical diagnostics and demonstrated its advantages as related to the state of the art in a particular subdomain. We focused on the following activities and fields:

- Assessing medicament efficacy in dermatology (Section 3.1).
- Assessing the objective progress of recovery in traumatology (Section 3.2).
- Quantifying applied force in forensic sciences (Section 3.3).
- Localizing exactly the cause of pain in physiotherapy (Section 3.4).
- Volumetric measurements of soft tissues (Section 3.5).
- Objective assessment of atopic dermatitis (Section 3.6).

The results obtained from the experiments, complemented with the methodologies used, are summarized in the relevant subchapters of this section. In order to provide a comprehensive overview of possible applications, we also included the studies already published; these studies already cite our original findings partially released through other articles, which contain more details about the experiment.

### 3.1. Assessing Medicament Efficacy in Dermatology

**Figure 10:**
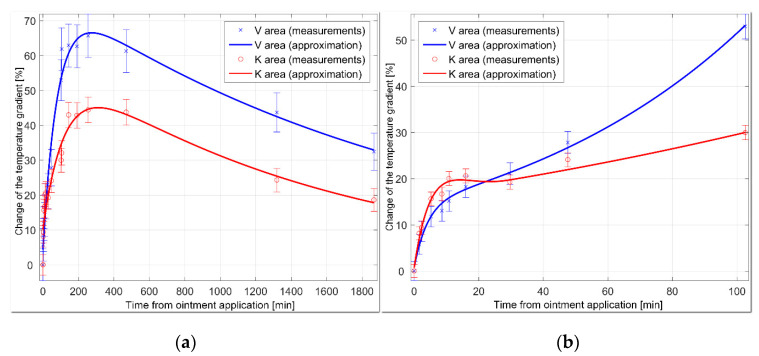
The development of the temperature gradient index in both areas during 31 h after the application of the ointments: (**a**) the overall progress of temperatures in both areas; (**b**) a detail of the first 100 min after the application of the drug [34].

The case study was performed on a subject suffering from allergy to hazelnut substances. The experiment started when red, itchy lesions appeared on one of the subject’s feet. The lesion-containing regions were highlighted with markers directly on the skin and then divided into two areas: K, where the Protopic 0.1% ointment was applied, and V, where a shea butter and coconut oil lotion was utilized. The patient was repeatedly scanned with the RoScan, and the average temperature gradient in both areas was evaluated by selecting the boundaries visible on the color layer of the 3D model. The results are shown in Figure 10.

The growth of the temperature gradient in the sector treated with Protopic 0.1% culminated earlier than in the shea butter and coconut oil zone, showing Protopic 0.1% to be the more effective medicament in this particular case.

It is important to stress that the series of experiments would not have been executable with a regular state-of-the-art method. At present, the clinical tests are usually performed on two groups of subjects, of which one is treated with the tested medicament, while the other is not; the overall progress of each subject’s symptoms (including, for example, redness, swelling, and itching) is evaluated by a coarse scoring system (usually not more than three grades). Such an approach then may lead to wrong conclusions if the selected group does not constitute a representative sample of the population (i.e., we do not know how the disease would develop without treatment in each patient), and its sensitivity is rather low.

The RoScan allows comparing the effects exerted by two different drugs on the same subject and even the same lesion, thus greatly increasing the reliability of the study because an identical spontaneous development is markedly more likely to be found in two parts of one lesion than in the lesions of two different patients.

This task is virtually impossible to implement via the current methods; moreover, the system’s sensitivity and selectivity enables the user to evaluate the progress of the treatment in great detail, as shown in Figure 10b. In the area K, the patient began to feel burning skin 2 min after the application of the ointments; the sensation culminated at 7 min following the medical intervention, and at 12 min the burning and itching in the area disappeared completely. In the area V, the itching did not change during the time. The patient’s report correlates with the response to the developing inflammation during the first minutes after the application of the drug: initially, the process appeared to be more intensive in the area K; here, however, the inflammation also regresses at a faster pace.

Another major advantage of the RoScan consists in that more quantitative parameters are available at the same time, while the current methods usually focus on a single item only; thus, in addition to the lesion temperature, the data relating to the color, reflectance, and roughness of the lesion surface and area are accessible.

As the thermal distribution of a patient’s surface temperature is affected by many environmental factors and also the subject’s pre-measurement activities, the measured index was established as a difference between the area’s average temperature and reference temperature, normalized by the value at the time when the dermatological medicaments were applied. Assessing the thermal difference between the reference point and the ROI eliminates environmental influences (it has been observed that the subjects’ previous activity, together with environmental effects, leads to an identical increase within the whole area). Normalization had been chosen due to the unequal distance of both areas from the edge of the body, i.e., a problem that causes differences in the absolute values of a temperature. This approach normalizes both values to the same scaled index and makes the areas comparable between each other. Further details concerning this experiment are provided in source [34].

### 3.2. Assessing Objective Progress of Recovery in Traumatology

**Figure 11:**
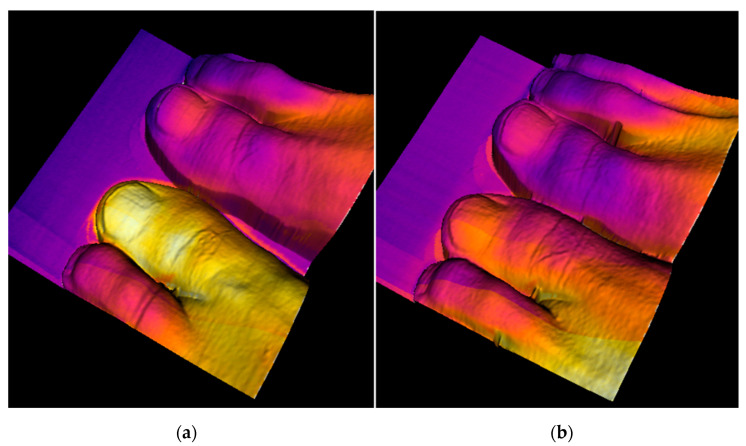
The injured toe captured 2 h (**a**) and 74 h (**b**) after the accident. The volume and the average temperature of the thumb appear to be reduced [35].

The experiment was performed with a subject having a bruised toe following a hit by a heavy object. Figure 11 contains images of the bruised toe immediately after the injury and then several hours later, with the affected area still clearly highlighted in the 3D thermogram.

In order to eliminate temperature fluctuations generated by external factors, which are non-negligible especially in the lower limbs, we choose the difference between the average temperatures of both toes as an objective index, since we assume that the impact of external influences will be comparable in both toes, and their thermal difference will then be almost zero in the physiological state.

**Figure 12:**
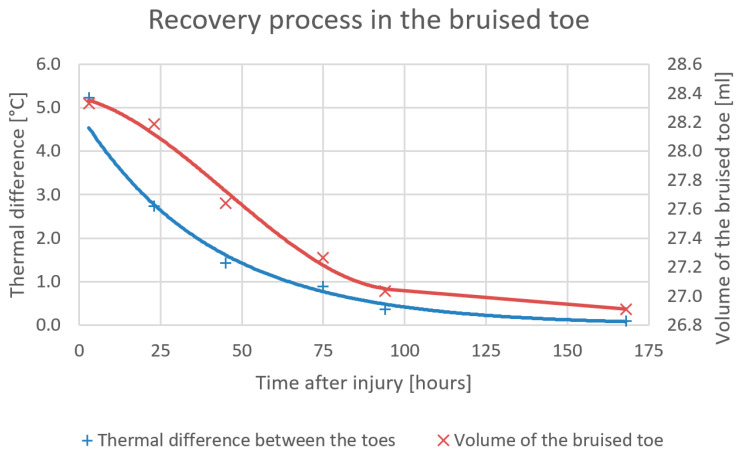
The progress curve relating to the average temperature in the region of the bruised toe, completed with an indication of the volume [35].

Figure 12 represents the development of the average temperature of the bruised toe; the actual process is expressed in relation to changes in the toe’s volume. The temperature decrease during the time exhibits an almost exponential trend: the swelling recedes slowly at the beginning but abates more quickly through the second and third days of recovery.

Importantly, the subject’s feelings of pain subsided already 74 h after the injury (Figure 11b); however, compared to the healthy toe, there was a still visible and measurable temperature difference (0.8 °C). Additional details on the experiment are outlined in source [35].

This scenario shows that the RoScan facilitates early detection of inflamed regions before they start to give pain or develop significantly; the system is thus capable of reducing the treatment and recovery time and cost. Unlike the current visual evaluation-based approach, the novel technique yields more accurate and objective data.

The relevant experiment was performed with five subjects suffering from the same problem at various degrees of intensity. In all of the cases, the thermal difference progressed similarly, with the maximum value corresponding to the bruise severity.

### 3.3. Quantifying Applied Force in Forensic Sciences

Another use for 3D thermography is in the evaluation of the force applied in violent crimes. Although the RoScan will probably be unable to determine the specific value of the force or the exact time of the actual act, it may beneficially allow comparison of two forces by examining their consequences.

**Figure 13:**
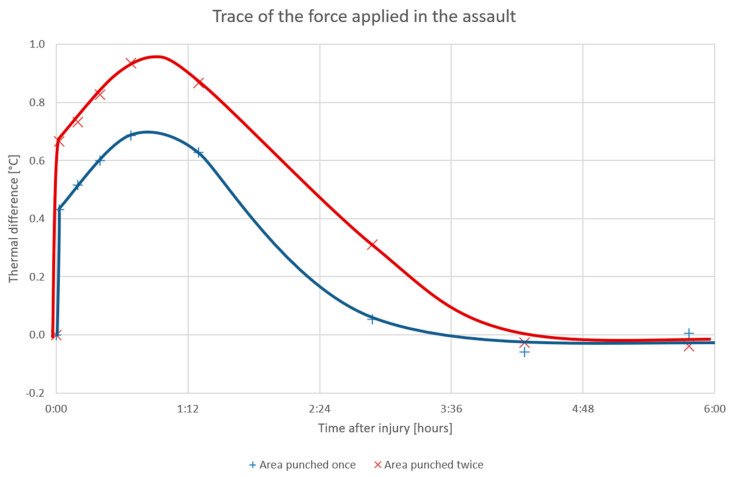
The thermal differences relating to the initial conditions and their progress during the first 6 h after the simulated assault; the subject was punched at t = 0:00.

In the simulation, the subject was punched with an imaginary attacker’s fist in the biceps, once and then twice, at two pre-marked places; the first was modeled by using a heavy pack of sand having a small, hard rubber ball attached to the bottom. The pack was invariably released from the same height, thus hitting the biceps with an identical force in each cycle. During the 6 h following the experiment, both areas were repeatedly scanned, and the temperature differences between the pre-marked locations and the pre-marked reference point on the shoulder were evaluated (Figure 13).

The temperature in both of the affected areas increased sharply at the exact time of the punch and then continued to grow during the first 50 min after the act. Interestingly, the measured temperature correlated with the number of the first hits, and the traces of the assault remained visible on the thermogram for more than 3 h following the punching. In this context, it should be noted that the “experimental punches” were very soft, and the subject had already stopped feeling pain 10 min later; such a progress stresses the high sensitivity of the method, even though in a real attack the signs would likely have been noticeable for markedly longer.

Conversely, we need to admit that the hits were aimed at a temperature-homogeneous area of the body, in which a uniform change caused by external influences can be expected. This type of results may not be generally achievable throughout the whole body; however, given that violent cases are often accompanied by a lack of evidence, such a record, too, has a useful potential.

### 3.4. Localizing Exactly the Cause of Pain in Physiotherapy

An important premise for selecting the right treatment in physiotherapy consists in ensuring the correct diagnosis of the cause of the pain [36]; however, this task is often very difficult to perform, given that at times we have to rely on only the feedback from a patient who cannot distinguish between different types of pain or accurately identify the relevant location [37].

In practice, DMTI is used where suitable, helping the examiners to reveal the source of inflammation and to determine the character of the problem (e.g., whether the cause rests in an articular block or muscular defects). The technique is nevertheless incapable of accurately localizing the center of pain and preserving an objective record similar to that produced by the RoScan.

Such records can be employed as follows: First, the ability to capture the current state of the patient objectively facilitates further comparison during his or her next clinical consultation; otherwise, if the changes are not large enough to be observable, the patient’s condition may be wrongly considered inalterable. The records also enable us to reliably assess the severity of the disease because the method is not as subjective as the others. Second, the thermal quantification (if possible) leads towards an assessment of the treatment suitability or medication effectiveness [35].

**Figure 14:**
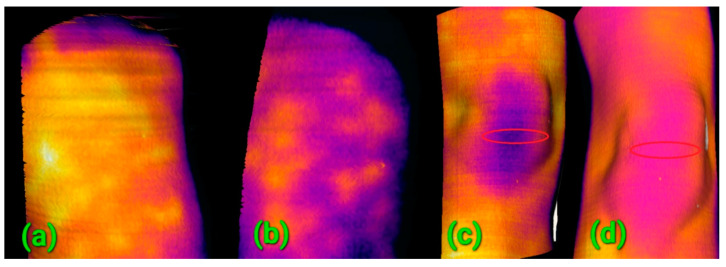
An upper limb affected by entezopathy of the deltoid muscle at (**a**) the first visit and (**b**) the second visit; the healing of the inflammation is clearly observable. The recovery following a surgery of ruptured knee ligaments at (**c**) the first visit and (**d**) the second visit; improved blood circulation is visible. Note: the regular hot spots in (**a**,**b**) are caused by cellulite.

In our study, several patients were repeatedly scanned with the RoScan during the recovery process following an injury or surgery. A comparison of the inter-visit 3D scans provided a proof of positive progress and facilitated the initial choice and definition of further treatment. In this context, for example, we can refer to the evidence of inflammation in a subject suffering from entezopathy of the deltoid muscle, revealed during the first examination (Figure 14a). The inflammation had recessed markedly before the second examination (Figure 14b), and improved blood flow in the patella area, accompanying the post-surgical recovery between the first (Figure 14c) and the second examinations (Figure 14d), is also evident. Positive progress was determined from the 3D models already after two days, when the condition was not yet evaluable by means of the common methods (which mostly exploit visual observation and manual examination).

The benefit of the RoScan in physiotherapy rests mainly in the exact localization capability; however, the device can also function as a quantification tool in cases where it is possible to suppress the external fluctuations that affect body temperature. Such a situation is presented in Figure 14a,b; the images show an area with a homogeneous temperature distribution where the external influences can be suppressed via comparison with a reference point nearby. In item 14ac, such normalization is difficult to achieve, and the user will thus benefit from the above-mentioned localization only.

### 3.5. Volumetric Measurements of Soft Tissues

**Figure 15:**
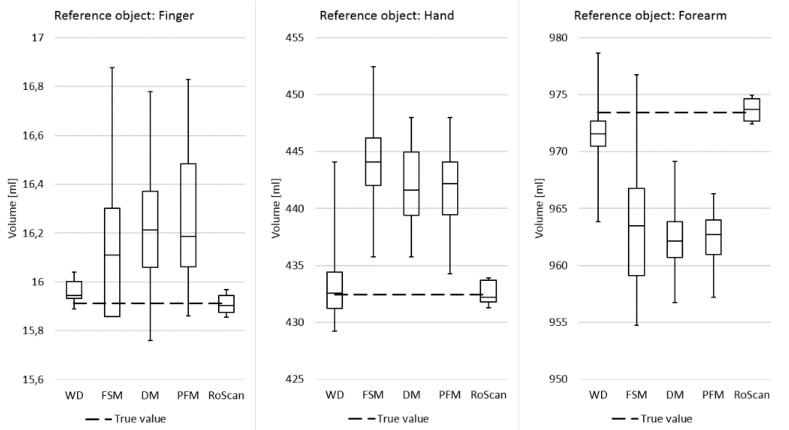
The RoScan compared with the circumferential techniques and the water displacement method implemented in a reference object having a known volume: the RoScan displayed the most accurate and repeatable performance [12].

Within this experiment, the current volumetric techniques were compared with the RoScan via measuring the volumes of reference objects (phantoms). When performing against the current gold standard, i.e., water displacement volumetry, the RoScan delivered volumetric measurements at higher accuracy and repeatability, as shown in Figure 15.

In terms of its parameters and capabilities, the RoScan embodies a faster, safer, and more accurate approach with very low operational costs; the system is especially valuable in specific cases where advanced ROI examination and high accuracy are needed. Such special volumetric cycles normally require the standard MRI or CT; however, the RoScan, if employed, reaches more accurate results at significantly lower costs [38].

As the RoScan is generally the most accurate method, it will allow measuring of even very small changes in the volume, which can enable us to discover pathological changes in a timely manner, well before they have developed significantly.

Additional details on the experiment can be found in source [12], and the current volumetric methods are compared between one another and with the RoScan in [39] and [38,40], respectively.

### 3.6. Objective Assessment of Atopic Dermatitis

There are many diagnostic methods for assessing atopic dermatitis (AD), varying from scoring systems (very subjective, insensitive, and providing low resolution) to sophisticated and expensive options [41].

In this context, DMTI is considered a relatively objective (and thus a potentially reliable) method, as it measures increased blood flow, which manifests itself almost identically in all patients [42]. The technique, however, is rarely used due to the low resolution and complicated standardization of the data that facilitate comparison between the results (the DMTI exact localization problem).

As mentioned above, the RoScan’s structure eliminates this difficulty, and thus it finds use in AD lesion assessment. The capability was demonstrated in the experiment, whereby an AD patient was examined via DMTI; nevertheless, the thermal data could not be causally linked to the real lesions. The relationship was then established only by using the RoScan and its ability to select the nonphysiological locations and to navigate in the color layer (Figure 8). The thermal information was then properly related to the AD lesions, and the progress was quantified to meet the goal of the experiment.

## 4. Discussion

The RoScan project, completely developed at Brno University of Technology, embodies a novel concept of using a robotic manipulator combined with several sensors producing data in different spectra. For medical applications, it appears to be advantageous to combine a laser scanner, a color camera (wavelength range between 390 and 750 nm), and a thermal imager (wavelength range from 8 to 14 µm); such a configuration is beneficial because the thermal changes commonly accompany the non-physiological states. This innovative method for multispectral 3D scanning brings several advantages compared to the state-of-the-art approaches.

Thanks to the projection of the spectral data onto the surface of the 3D model via an independent localization system, the process of registering the 2D images is robust and independent of the scanned object. The procedure is applicable to any smooth and homogeneous surface or texture, thus being especially valuable in merging several thermal images into a large one if there is a lack of reliably detectable registration landmarks.

Another major benefit of independent localization lies in cross-spectral image registration (color vs. thermal). With this method, one can reach the same resolution in both the thermal and the color layers, even if the native resolutions of the sensor differ significantly. Moreover, both layers are properly aligned to the 3D model and to each other; importantly, the uncertainty is exactly specified and equal in the entire dataset.

Advancement beyond the possibilities of the state-of-the-art is recognizable also within 3D scanning; unlike many of the current, commercially available 3D methods, the RoScan allows each point of the captured model to exhibit an equal, absolute spatial accuracy independent of the scanned scene and lacking cumulative deviations. Additionally, the accuracy is precisely quantifiable from the parameters of the manipulator and sensor, bringing a major advantage in precise measurements, where the uncertainty must be specified in an exact manner. Most of the current 3D scanners are not capable of such operations [43,44].

The RoScan offers a considerable potential in those medical applications where objective quantification plays the key role. The system quantifies inflammatory processes inside the human body; several pilot case studies described in this paper showed that using the RoScan to quantify inflammation is feasible when the inflamed region remains close to the skin, has sufficient “strength” to influence the skin temperature, and ideally appears in an area where the temperature distribution is as homogeneous as possible, enabling us to find an index with the potential to eliminate the environmental factors.

The RoScan-based assessment of medicament efficacy in dermatology showed the possibility of comparing the impacts of two different drugs on the same subject and even the same lesion, an objective virtually unachievable via the current methods. Standard clinical tests are usually carried out on two groups of subjects, of which one is treated with the tested medicament, while the other is not; such an approach then may lead to wrong conclusions if the selected group does not constitute a representative sample of the population, and its sensitivity is rather low (meaning that only major progress is distinguishable).

A traumatology-related study discussing the assessment of objective progress in recovery pointed out that the RoScan enables early detection of inflamed regions before they start to give pain or develop significantly, stressing also the facts that the system is thus capable of reducing the treatment and recovery time and cost.

Moreover, the technique embodies a valuable tool to yield an objective record of the current state of a disease; producing such records is a common problem of present medicine, which intensively relies on the personal memory of the clinician.

Another research subdomain where the RoScan reaches beyond the state of the art lies in investigating the quantification of applied force: the system proved to be highly sensitive, with the traces of a physical assault remaining visible on the thermogram for more than 3 h after the violent act, even though the actual pain caused by the punching disappeared in only 10 min. The measured temperature correlated with the number of punches received.

In physiotherapy, the RoScan delivered objective evaluation of the treatment effects, documenting objectively blood circulation improvement after an invasive surgery.

Finally, the tool has been verified as suitable for soft tissue volumetry, bringing superior accuracy and repeatability. An additional benefit is the possibility to select the edema only, stemming from the ability of the thermal layer to distinguish the edema from a healthy tissue. Neither of the achievements is obtainable via the current volumetric methods.

At this point, however, it is necessary to emphasize that 3D thermography remains a mere tool requiring expert assessment by a doctor. The RoScan does not aim to offer automatic detection and localization of inflammation; rather, the device is designed to embody a source of objective data that will allow clinicians to decide and quantify better and more correctly than if such data are lacking. Areas with higher temperatures are not always inflamed; often the effect expresses an anatomical constitution (e.g., when the arteries lie closer to the skin). A significant impact is generated also by external influences, including the ambient temperature, the patient’s previous activity, and current footwear or clothing. Although the effect of these elements can be limited in many cases by choosing a suitable relative index, this approach is not applicable generally, meaning that the 3D thermogram will still have to be interpreted and evaluated by a medical doctor, who nevertheless may not be as experienced as a physician diagnosing without the thermogram.

## 5. Conclusions

Generally, precise 3D thermography tools, such as the RoScan, can yield major benefits in various healthcare domains. The relevant instruments facilitate early detection of inflamed areas, the reason being that their sensitivities surpass those of the commonly used methods, which are mostly based on visual observations [45,46,47]. The precise 3D thermography as performed by the RoScan addresses three main limitations of Digital Medical Thermal Imaging (DMTI), discussed in the present scientific literature: the exact localization problem, the resolution vs. range problem, and quantification impossibility. In this context, DMTI has been characterized as having a substantial potential if the drawbacks were resolved.

The RoScan system eliminates all of these issues, thus opening new possibilities for thermal imaging in medicine. Several pilot case studies confirmed the benefits introduced by the RoScan into diverse subdomains of healthcare, where the advanced concept overcomes the limits inherent in the other state of-the-art methods. This scanning approach appears to be particularly beneficial within medical efficacy assessment, dermatology, traumatology, and physiotherapy, where it facilitates objective monitoring of the recovery progress. In all of these areas, the novel multispectral 3D method can be advantageously adopted as an objective quantification tool to fill the void in the set of options available for the given purpose.

More concretely, the RoScan is especially useful when the common symptoms have not yet fully developed; however, multispectral 3D scanning in general finds application also when the symptoms are already visible. Importantly, the capability of preserving the exact state of the patient’s body (to allows comparison during the next visit at the clinician) leads to objective evaluation of the progress of the disease.

The positive results of the initial case studies presented herein pointed out the areas where the opportunity to enhance the state of the art is potentially high. In future projects, we will focus predominantly on these domains, and we will attempt to transfer the proposed technology into everyday medical practice. Although it will probably be necessary to modify the system for the given applications, this task will not be problematic, especially due to the fact that the entire system was fully developed at our department and is thus completely controlled by the authors of the paper. Major portions of the research will be dedicated to the maximum simplification and automation of the scanning process because if the system is to be practically usable, it must offer easy operability without expert knowledge and significant time intensity.

The system is convenient for not only medicine but also technology and science: thermal 3D scanning procedures are usable in, for example, electrical engineering to ensure thermal analyses centered on the efficiency of electrical components [48]; civil engineering to monitor thermal loss in materials [49]; mechanical engineering to investigate mechanical strain [50]; and rescue robotics or experimental biology to evaluate the indispensable life functions [51,52,53].

Moreover, if we also take into account the fact all algorithms are universal and thus make it possible to easily replace the color and thermal camera with another sensor of a different spectral range, the usability of the RoScan system will expand even more significantly.

## Figures and Tables

**Table 1:** The key parameters.

Parameter	Value
Positional accuracy of model points (3 σ).	0.12 mm
Scanning speed	10 mm/s
Scanning trajectory	flexible, programmable
Measured values	position, color, temperature, roughness
Operational cost	approximately 0.1 EUR per model
Price of a device	approximately 30,000 EUR (or less)
Data storage format	internal
Export formats for other software	PLY, PTS, XYZ
